# Internet publicity of data problems in the bioscience literature correlates with enhanced corrective action

**DOI:** 10.7717/peerj.313

**Published:** 2014-04-03

**Authors:** Paul S. Brookes

**Affiliations:** Department of Anesthesiology, University of Rochester Medical Center, Rochester, NY, USA^*^* The author wishes to emphasize that the data collection for this research was conducted outside the boundaries of his position as a University of Rochester faculty member. The author assumes full responsibility for this work, and his affiliation with the University of Rochester does not represent an endorsement of this work by the institution.

**Keywords:** Retraction, Correction, Erratum, Image manipulation, Social media, Science publishing

## Abstract

Several online forums exist to facilitate open and/or anonymous discussion of the peer-reviewed scientific literature. Data integrity is a common discussion topic, and it is widely assumed that publicity surrounding such matters will accelerate correction of the scientific record. This study aimed to test this assumption by examining a collection of 497 papers for which data integrity had been questioned either in public or in private. As such, the papers were divided into two sub-sets: a public set of 274 papers discussed online, and the remainder a private set of 223 papers not publicized. The sources of alleged data problems, as well as criteria for defining problem data, and communication of problems to journals and appropriate institutions, were similar between the sets. The number of laboratory groups represented in each set was also similar (75 in public, 62 in private), as was the number of problem papers per laboratory group (3.65 in public, 3.54 in private). Over a study period of 18 months, public papers were retracted 6.5-fold more, and corrected 7.7-fold more, than those in the private set. Parsing the results by laboratory group, 28 laboratory groups in the public set had papers which received corrective action, versus 6 laboratory groups in the private set. For those laboratory groups in the public set with corrected/retracted papers, the fraction of their papers acted on was 62% of those initially flagged, whereas in the private set this fraction was 27%. Such clustering of actions suggests a pattern in which correction/retraction of one paper from a group correlates with more corrections/retractions from the same group, with this pattern being stronger in the public set. It is therefore concluded that online discussion enhances levels of corrective action in the scientific literature. Nevertheless, anecdotal discussion reveals substantial room for improvement in handling of such matters.

## Introduction

It is widely believed (with somewhat little proof) that the scientific process is inherently self-correcting, and the integrity of the scientific record has always been very important. However, recently the issue of data integrity in published scientific literature has received an unprecedented level of attention both in traditional media outlets such as journal editorial pages (Mole, 2012; Bosch, 2013), as well as new media such as blogs and social media websites. A new sub-field has emerged to study the processes that underlie corrections to the scientific literature, and to highlight patterns in the mechanisms of corrective action.

Notable findings in this area include an apparent increase in the rate of scientific retractions in recent years (Steen, Casadevall & Fang, 2013), as well as conflicting reports on whether scientific misconduct is a predominant reason for retractions (Fang, Steen & Casadevall, 2012; Grieneisen & Zhang, 2012). With regard to the latter, there is some indication that retraction notices may not be a reliable source of information on underlying causes (Fang, Steen & Casadevall, 2012; Resnik & Dinse, 2013). Furthermore, although there appears to be a positive correlation between journal impact factor and willingness to retract manuscripts (Fang & Casadevall, 2011), a recent trend toward “mega-corrections” in high impact-factor journals has also been noted (Oransky & Marcus, 2012), suggesting that methods for dealing with problematic data are still evolving.

**Blog Roll**
http://abnormalscienceblog.wordpress.com: *Run by former German scientist Joerg Zwirner. Closed in late 2012 due to personal issues.*
http://blog.goo.ne.jp/11jigen: *Anonymous Japanese blogger Juuichi Jigen, who runs numerous websites, each alleging misconduct by individual scientists.*
http://copy-shake-paste.blogspot.com: *Run by German scientist Debora Weber-Wulff, dealing mostly with plagiarism in academic documents authored by European politicians.*
http://retractionwatch.wordpress.com: *Run by two US science journalists. Covers retractions and surrounding issues. Anonymous comments often include misconduct allegations.*
http://www.science-fraud.org: *Run anonymously by this article’s author, reporting on alleged data problems. Site closed by legal threats in Jan 2013.*
http://www.pubpeer.com: *Run by anonymous junior scientists. Permits users to anonymously comment on any paper in the PubMed database.*
http://www.ncbi.nlm.nih.gov/pubmedcommons/: *NCBI’s comment system, currently in beta testing, requires commenters to use a real identity.*

As a part of the rapidly developing media landscape in this area, several web-sites have emerged (see “Blog Roll” inset), for readers to post and discuss problematic images and other data, often anonymously. However, this has been met with some resistance from established science media outlets (Mole, 2012; Parak et al., 2013), and several of these sites have been subjected to legal threats (Couzin-Frankel, 2013),^1^1http://www.popehat.com/2013/04/11/ their proprietors accused of vigilantism (Mole, 2012), and in some cases shut down altogether (Couzin-Frankel, 2013).

With the escalating adoption of social media techniques by science activists, it is critical to ask whether such public discussion of data integrity actually has any effect? Although it is widely assumed that such efforts may enhance the motivation of journals, authors or institutions to take corrective action, this hypothesis has not been rigorously tested. Herein, analysis of nearly 500 papers with alleged data problems reveals a significant corrective effect of enhanced publicity.

## Methods

From July to December 2012, the author of this study was the anonymous proprietor of a blog site (see inset). During this time emails were received to the site, alleging data integrity problems in published journal articles, mostly in the life sciences. These emails were received from individuals using anonymous email accounts, to an anonymous email account protected by two-factor authentication (G-mail), to protect the identity of communicants. Furthermore, all research materials were stored on an encrypted hard disk. Some 274 published papers were documented in blog posts, with specific illustration of the alleged problems, plus relevant background information (e.g., whether authors had other papers retracted/corrected).

In January 2013 legal threats forced the prompt closure of the site, but anonymous submissions continued, and a large quantity of prepared material was left unpublished. This activity yielded a further 223 papers with documented problems, received between November 2012 and January 2013. These papers remained in a private collection.

The 497 papers (274 public + 223 private) all met the same basic criteria for the definition of problem data (i.e., documented allegations by an anonymous correspondent, and confirmation of such by myself, using forensic tools such as droplet plug-ins for Adobe Photoshop™ provided by the US Office of Research Integrity^2^2http://ori.hhs.gov/droplets). Anonymous correspondents had also CC’ed journals, authors’ host institutions and funding agencies. As such, there was no selection bias present between the public and private papers: all would have eventually been publicized if circumstances had developed differently.

For each paper, the following parameters were collated into a database: (i) PubMed ID, (ii) Journal, (iii) Year, (iv) Volume, (v) Page #, (vi) List of problematic data panels, (vii) 5 year impact factor of the journal (2008–2012, ISI Journal Citation Reports), (viii) Outcome. Outcomes were classified into three groups: retraction, publication of an erratum or corrigendum, and no action so far (December 2013). In the case of errata/corrigenda, only those dealing directly with the subject of the questioned data were counted. In addition, only retractions occurring within the time-frame of this study (July 2012 to December 2013) were counted, although it should be noted that some journals do not give reasons for retraction, so attribution of a retraction to a precise cause was not always possible.

The majority (∼75%) of problems encountered were apparent inconsistencies in western blotting data (undisclosed splicing, or apparent re-use of bands or blots to represent different experimental conditions), with the remainder relating to apparent re-use of light/fluorescent/electron microscopy images, apparent re-use of text, and apparent re-use of FACS histograms to represent different experimental conditions. Most cases involved data within a single paper, but in a small percentage of cases data appeared to be re-used between papers originating from the same laboratory group.

Due to the sensitive nature of its content, the full data set for this study comprising the list of publications, including those for which no action was taken, cannot be provided. However, a de-identified (blinded) version is provided in an accompanying online supplement. Where appropriate, statistical differences between groups were determined using ANOVA, and data are presented as means ± standard deviations with 95% confidence intervals.

## Results

Properties of the public and private paper sub-sets are shown in Table 1. Overall the sets exhibited no differences in number of problematic data panels per paper, or in the 5 year impact factor of the journal they were published in. There was a trend toward papers in the private group being slightly older, although the reasons for this are not fully understood.

**Table 1:** Properties of papers in the public and private sub-sets. Means ± standard deviations, with 95% confidence intervals where appropriate.

	Public	Private
# papers	274	223
# Retractions (%)	16 (5.8)	2 (0.9)
# Corrections (%)	47 (17.2)	5 (2.2)
# of problematic data panels/paper *(95% CI)*	2.3 ± 1.7 *(2.1–2.5)*	2.5 ± 1.5 *(2.3–2.7)*
5 yr. journal impact factor *(95% CI)*	9.3 ± 8.5 *(8.3–10.3)*	8.7 ± 7.1 *(7.7–9.6)*
Publication year	2007.5 ± 4.3	2004.8 ± 4.1
Total # of laboratory groups	75	62
# of problematic papers/group	3.65 ± 3.61 *(2.79–4.42)*	3.54 ± 5.16 *(2.26–4.82)*
# of laboratory groups with action on papers	28	6
Papers with action, as % of those flagged for a given laboratory group *(95% CI)*	62.4 ± 31.5 *(50.8–74.1)*	26.8 ± 26.1 *(5.9–47.7)*

For primary outcomes, the public set exhibited a 6.5-fold fold higher rate of retractions, and an 7.7-fold higher rate of corrections, versus the private set. Combined, 23% of the publicly discussed papers were subjected to some type of corrective action, versus 3.1% of the private non-discussed papers. This overall 7-fold difference in levels of corrective action suggests a large impact of online public discussion.

The number of laboratory groups represented was similar between the public and private sets (75 and 62 respectively), as was the average number of identified problematic papers per laboratory group (3.65 public versus 3.54 private). However, despite these similarities, 28 laboratory groups in the public set had at least one paper with corrective action taken, versus only 6 laboratory groups in the private set. Furthermore, corrective actions appeared to be more *clustered* in the public set. For laboratory groups in this set with corrected/retracted papers, such actions extended to cover almost 2/3 of those initially flagged as problematic (62%). In contrast, for laboratory groups in the private set with corrected/retracted papers, such actions covered little over 1/4 of those initially flagged as problematic (27%). This suggests that corrective actions in the private set took place on a more individualized basis, with more clustering of corrective actions in the public set perhaps being a direct consequence of greater publicity.

Within the public set alone, parsing the papers into outcome groups (Table 2) indicated a trend toward more problematic data panels per paper and lower journal impact factor in the retracted group. In addition a trend toward more recent publication year was seen in both retracted and corrected papers, relative to those for which no action was taken. However, the small sample size (particularly in the retracted paper group) did not permit strong conclusions to be drawn regarding these trends.

**Table 2:** Selected properties of papers in the public set, broken down by outcome. Means ± standard deviations, with 95% confidence intervals where appropriate.

	Retracted	Corrected	No action
# papers^a^	16	47	212
Problematic data panels/paper *(95% CI)*	3.1 ± 38 *(2.5–4.2)*	2.2 ± 1.4 *(1.8–2.7)*	2.2 ± 1.4 *(2.0–2.4)*
5 yr. journal impact factor *(95% CI)*	6.9 ± 3.7 *(5.2–8.6)*	10.0 ± 8.6 *(7.5–12.4)*	9.3 ± 8.7 *(8.2–10.5)*
Publication year	2008.3 ± 4.4	2009.3 ± 3.0	2007.1 ± 4.4

**Notes.**

a Total = 275, not 274 as expected, since one paper was corrected and subsequently retracted.

## Discussion

The primary finding of this study is that online discussion of problematic data is correlated with an approximately 7-fold greater likelihood of either correction or retraction of a paper. This is the first study of its type, and the result should serve as an impetus to encourage further engagement of new media, to push for greater integrity in the scientific literature. In addition, the result suggests that institutions charged with addressing such problems do pay attention to online publicity.

In addition, an association was observed between publicity and clustering of corrective actions. Similar numbers of laboratory groups were represented in each set, and the number of papers per laboratory group initially flagged as problematic was also no different. Together, these indices suggest that opportunities for corrective action to take place in a clustered manner (i.e., acting on several papers at once) were the same between the public and private paper sets. Nevertheless, more clustering (defined as percentage of total papers flagged for a given laboratory group eventually being acted on) was observed in the public set, while corrective actions in the private set appeared to take place more on an individual paper basis. It is possible that publicity was a factor driving this difference—i.e., institutions may be more willing to take action on papers if they are aware of other problem papers by the same laboratory group, via public discussion forums such as those mentioned earlier. In contrast, if problems identified in papers remain in the private domain, communicated only on an individual basis, then institutions may not see *the big picture*, and be less willing to take action.

The average time from publication to retraction in this study was 4 years, which agrees with previous estimates (Steen, 2011). However, the observed trend toward greater corrective action for more recently published papers is somewhat counter-intuitive, since it might be expected that newer papers have been read and scrutinized less. This trend could be due to evolving literature consumption patterns among scientists, such that newer papers are more readily available and so read and scrutinized more. Alternatively it may reflect the US Office of Research Integrity’s 6 year statute-of-limitations on investigating allegations of misconduct, such that there is less pressure to correct older papers, or insufficient evidence in the form of backup data to prove/disprove any allegations. Finally, this trend toward more corrections in the recent literature could be due to a reported recent uptick in the levels of research misconduct (Steen, Casadevall & Fang, 2013; Steen, 2011).

Regardless of the age of corrected papers, it should be emphasized that the overall levels of corrective action observed in this study are still rather low, at 23.0% in the public group and only 14.1% for the complete set of 497 papers. One reason for this (and an important caveat of this study) could be the short study duration of 18 months, such that insufficient time has passed for thorough investigations by journals and institutions. Thus, it will be interesting to revisit these data in future, to see if more papers are corrected. The possibility cannot be ruled out that, given sufficient time, papers in the private set will *catch-up* to those in the public set, although this appears unlikely given current margin between these sub-sets.

Another reason for low overall levels of corrective action could be that the alleged problems in these papers are ill-founded and do not warrant action. It is almost impossible to gauge the magnitude of this problem because the current system of reporting on data integrity only publicizes actionable findings. Journals and institutions often conduct investigations in private and do not disseminate results if no wrongdoing is found. As such, there could be a large number of papers for which a no-fault outcome has been assigned, but this will never be known publicly. Furthermore, counteracting such under-reporting of ill-founded allegations, there are also likely to be cases in which allegations are sound, but retraction or correction notices are insufficiently detailed to indicate this. Thus, even in the case of sound allegations it can often be difficult to provide a solid link between a specific problem in a paper and a course of action taken on it by the journal.

**Anecdotal evidence of a corrective system in need of improvement…**
*Case 1*: *I contacted a journal anonymously to highlight problems in 5 papers. 15 subsequent emails to the journal, several editorial board members, and the governors of the scientific society that oversees the journal, all failed to elicit a single response, even to acknowledge correspondence. Refusal to communicate is contrary to guidelines of the Committee on Publication Ethics (COPE) which the journal is a member of.*
*Case 2*: *I reviewed a paper and found fabricated data. The journal rejected the paper, and subsequently it was published in a different journal with some problem data still present. The editor at the new journal knows about the previous rejection for reasons of data fabrication, but refuses to take up the matter with the authors unless I am willing to have my real name revealed as an accuser. I refused, because the lead author is on a panel that reviews my grant proposals.*
*Case 3*: *Two multi-panel figures were duplicated in their entirety, including figure legends and descriptive text, in two papers in different journals, submitted a week apart. Both journals permitted authors (who have retracted 2 other papers for acknowledged misconduct) to issue a correction, merely stating the data were the same. COPE guidelines, to which both journals subscribe, are quite clear regarding dual submission of data to more than one journal.*
*Case 4*: *I reported on fabricated data in a supplementary file. 3 months later a blog commenter (whose IP address resolved to the city of the lead author) claimed the report was incorrect, and demanded its removal. Coincidentally, that same day the journal website had posted a new supplemental data file, with the problem data replaced, but no correction notice. I contacted the journal, but more than a year later they have not acknowledged the correction took place.*

Another reason for low levels of corrective action is suggested by anecdotes (see inset) indicating that journals and other institutions may not wish to engage in dealing with such matters. Many journals do not respond to allegations from anonymous correspondents as a matter of policy, and while there are several reasons for this (e.g., not wishing to allow scientific competitors to sabotage rivals’ work), it is clear that journals do have some leeway in determining whether to respond to anonymous correspondents. Aside from the issue of anonymity, these anecdotes are diagnostic of a corrective system that is far from perfect. While it is beyond the scope of this manuscript to speculate on ways to improve the corrective system in the scientific literature, recent developments such as PubPeer and PubMed Commons are seen as steps in the right direction, toward universal and open post-publication peer review.

With discussions ongoing in the scientific community regarding post-publication peer review, there appears to be little agreement overall on the issue of anonymity. While anonymity is often beneficial for junior scientists (who may for example fear repercussions when raising questions about a senior scientist’s work), a purely anonymous system is also open to abuse (e.g., sabotage of colleague’s work). A moderated discussion system may help to avoid such abuses, although in the current fiscal climate it is unlikely that sufficient funds exist to pay for moderators, who would necessarily have to be highly trained in scientific sub-fields.

Some other important caveats to this study are as follows: (i) The study is limited by a somewhat small sample size, particularly for the retracted group of papers. (ii) The data for the study came from a limited number of anonymous correspondents and concerned mostly problems with image manipulation in life-sciences papers. As such, it is not clear if the patterns observed herein are generalizable to the scientific literature at large. (iii) The study was not prospectively designed, and although every care was taken to conduct it in an ethically sound and unbiased manner, the research was conducted by the author as a private citizen and therefore fell outside of university institutional review board (IRB) oversight. While it is not immediately obvious that such research would even fall under the topic of human subjects research, the anonymity of correspondents reporting on papers was strictly maintained, and to date all remain anonymous to the author. (iv) Every effort was made to ensure that problems identified were communicated adequately to the appropriate parties, but this could not be verified for every single paper. In some cases, the only evidence supporting knowledge of a problem by a journal or institution, was the word of an anonymous email correspondent. Attempts to verify such information were rendered difficult by non-disclosure policies surrounding ongoing investigations, and this information is likely impossible to verify completely. (v) While the author has made efforts to make the data set available to the fullest extent possible during peer review, clearly these data are of a sensitive nature, and as such it is unlikely that the study can be reproduced independently. (vi) There are likely to be unknown and uncorrected factors that were different between the public/private paper sets. These could include subtle differences in scientific sub-field between the sets (e.g., cancer vs. neurology) or the precise make-up of sub-fields or nationalities to which the anonymous correspondents belonged in each set. While it is unlikely such factors will ever be fully resolved, the large difference in primary outcomes between papers discussed online and those not (i.e., 7-fold greater levels of corrective action), suggests this result is unlikely due to such factors entirely.

In summary, the current study shows that publicity surrounding issues of problematic data is correlated with greater levels of subsequent actions to correct the scientific record. Nevertheless, anecdotal evidence suggests there is substantial room for improvement in the standards for dealing with such issues at the institutional and publisher levels.

## Supplemental Information

10.7717/peerj.313/supp-1 Supplemental Information 1 De-identified data set Spreadsheet contains full list of alleged data problems for 274 papers in the public set and 223 papers in the private set, with all identifying information removed.
